# Clinical Decision Making: Sickle Cell Crisis with Acute Chest Syndrome

**DOI:** 10.5070/M5.53173

**Published:** 2026-10-31

**Authors:** Ryan Adkins, Aubrey Bethel, Sara Dimeo

**Affiliations:** * Creighton University East Valley Arizona, Department of Emergency Medicine, Chandler, AZ

## Abstract

**Audience:**

This clinical decision-making case (CDM) is intended for emergency medicine residents (interns, juniors, seniors).

**Introduction:**

Acute chest syndrome (ACS) is a life-threatening complication of sickle cell disease and can be a common cause of ICU admission in these patients. Emergency physicians must rapidly identify, diagnose, and treat ACS to prevent deterioration. Acute chest syndrome is the most prevalent pulmonary disorder in individuals with sickle cell.^1^,^2^ Approximately 50% of patients with sickle cell disease will experience more than one episode of acute chest syndrome, with 78% of episodes secondary to vaso-occlusive pain episodes in adults. It is the most common cause of death in individuals with sickle cell, accounting for almost 25% of all deaths.^3^

**Educational Objectives:**

By the end of this clinical decision-making case, learners will be able to: 1) demonstrate familiarity with a case, 2) identify focused historical and physical exam findings in ACS, 3) develop a differential diagnosis for chest pain in a sickle cell patient, 4) select and interpret appropriate diagnostic studies, 5) initiate ED management including oxygen, antibiotics, and pain control, 6) recognize when to consult hematology and consider transfusion, 7) appropriately disposition the patient and communicate a transition of care, and 8) explain the pathophysiology of ACS.

**Educational Methods:**

The development of this case was grounded in Kolb’s experimental learning theory.^4^ This process includes four stages: experience, reflection, conceptualization, and experimentation to solidify learning of skills and concepts. This case was delivered in three phases to encompass all stages of Kolb’s learning theory: flipped classroom instructional video simulated practice and role play and formal assessment (the scoring checklist). This case uses a structured oral board simulation based on American Board of Emergency Medicine’s (ABEM) Clinical Decision-Making format (CDM).^5^ First, the residents were asked to watch a video from the ABEM website on how to perform the CDM model. Immediately afterwards, they were asked to reflect on whether they had had previous experience with this model of teaching and to fill out the survey. Finally, on a simulation didactic day, residents were randomized into groups of three to four. An emergency medicine attending administered the case over 15 minutes to one resident in the group, while the other group members observed over 15 minutes, with 25 minutes to debrief. Learners answer standardized prompts in a timed, non-roleplay format, enhancing diagnostic reasoning and management planning. The attending and a separate resident then scored the participant using the provided scoring. Immediately afterwards a brief debrief was held and the scores were reviewed. The method aligns with constructivist learning theory and supports deliberate practice.

**Research Methods:**

Residents were consented and then randomly assigned a study number, and they filled out pre- and post- confidence level surveys based on their experience with the clinical decision-making format. A Likert scale was used from 1–5, 1=Not at all confident, 5 = Very confident. After completing the in-person case session, the residents were asked to fill out a post-confidence level survey. To assess whether the intervention led to statistically significant changes in confidence levels, we conducted a paired t-test across the pre- /post- groups.

**Results:**

Thirteen residents consented to and participated in the study by completing both the pre-and post-confidence level survey. Eight of the residents were PGY 1, and five were PGY 2. Between both groups, the confidence level improved overall from 1.86 pre- to 2.92 post (*P*=0.005). PGY 1 residents had a lower confidence level, starting out at an average of 1.5 with improvement to 2.63 (*P*=0.026), compared to PGY 2’s who started out at 2.4 and improved to 3.4 (p=0.089). The PGY 2 improvement was not statistically significant.

**Discussion:**

The structured format allowed for targeted feedback and consistent assessment. Residents appreciated the immediate feedback and felt more confident in their preparation with the oral boards clinical decision-making format. Interns had the greatest improvement in confidence levels when compared between residency classes. This could represent PGY 2’s increased clinical experience. This format was done in the first months of residency as well, and it would be interesting to see if there was improvement from the beginning of the year to the end in the intern class just from patient exposure. Our program does not currently have PGY 3’s but repeating this study in the future would be beneficial to compare and see if the largest gains are in the intern and junior residents with less clinical experience.

**Topics:**

Acute chest syndrome, sickle cell disease, hypoxia, chest pain, exchange transfusion, incentive spirometry, ICU admission, ABEM oral boards, antibiotic management, emergency medicine simulation.

## USER GUIDE

**Table:** 

List of Resources:	
Abstract	1
User Guide	3
For Examiner Only	5
Certifying Exam Assessment	13
Stimulus	15
Debriefing and Evaluation Pearls	23

**Learner Audience:**

This CDM is intended for interns and junior and senior residents.

**Time Required for Implementation:**

Case: Clinical decision-making cases are 15 minutes by American Board of Emergency Medicine (ABEM) standard. Debriefing: 5–10 minutes per case.

**Recommended number of learners per instructor:**

1–4 residents

**Topics:**

Acute chest syndrome, sickle cell disease, hypoxia, chest pain, exchange transfusion, incentive spirometry, ICU admission, ABEM oral boards, antibiotic management, emergency medicine simulation.

**Objectives:**

By the end of this clinical decision-making case, learners will be able to:

1. Demonstrate familiarity with a clinical decision-making case.
2. Identify focused historical and physical exam findings in ACS.
3. Develop a differential diagnosis for chest pain in a sickle cell patient.
4. Select and interpret appropriate diagnostic studies.
5. Initiate ED management including oxygen, antibiotics, and pain control.
6. Recognize when to consult hematology and consider transfusion.
7. Appropriately disposition the patient and communicate a transition of care.
8. Explain the pathophysiology of ACS.

### Linked objectives, methods and results

The CDM case format gives learners a realistic, time-pressured way to practice clinical reasoning using structured prompts that mirror ABEM-style oral boards. Learners move stepwise through targeted questions such as:

- What additional history do you want?
- What are your leading diagnoses and why?
- What tests will you order right now?
- How will you manage the patient in the next 5 minutes?
- What will you do if the patient worsens?
- What is your disposition and contingency plan?

Each objective is met by deliberately linking prompts to core actions: focused history-taking and physical exam to identify high-risk features, building and prioritizing a differential diagnosis, selecting diagnostics that confirm or exclude life-threatening conditions, initiating evidence-based treatments and reassessing response, and making safe disposition decisions with clear escalation triggers, consults, and return precautions. This structure makes learners verbalize not only *what* they would do, but *why*—reinforcing clinical prioritization, anticipatory thinking, and board-ready communication.

This format was selected to reinforce real-world prioritization and decision-making using deliberate practice and constructivist learning principles. Learners apply knowledge in context, receive immediate feedback, and refine clinical judgment in a high-stakes simulation.

### Recommended pre-reading for instructor

- American Board of Emergency Medicine. Clinical decision-making oral board examination format. Published 2024. Accessed January 21, 2026.
- *Rosen’s Emergency Medicine: Concepts and Clinical Practice*. 9th ed. Elsevier; 2018:Chap 112. Edited by Robert M. Walls, Robert S. Hockberger, and Marianne Gausche-Hill.
- *Tintinalli’s Emergency Medicine: A Comprehensive Study Guide*. 9th ed. McGraw Hill; 2020:Chap 236. Edited by Judith E. Tintinalli, O. John Ma, David M. Yealy, et al.
- EM:RAP. C3: Sickle Cell Crisis [podcast]. Published 2024. Accessed January 21, 2026. https://www.emrap.org/episode/shockasthmaand/annalsof

### Results and tips for successful implementation

Residents were first given an instructional video through the ABEM website on how to perform the CDM format,^5^ and then asked to reflect on similar formats that they have performed in the past. On didactic day, they were organized into groups ranging from three to four composed of medical students, and PGY 1 and PGY2 residents. One resident was in the “hot seat” answering questions while one resident was helping keep score, and all the other residents observed. The resident in the “hot seat” was scored by the attending and one other resident using the scoring rubric, and then 25 minutes of debrief occurred immediately afterwards going through each point on the rubric. Thirteen residents consented to and participated in the study by completing both the pre-and post-confidence level survey.

The instructors reviewed the case prompts and expected answers before the session so they could guide the learner smoothly through the scenario and keep the pacing aligned with an ABEM-style format. They had a printed copy of the critical actions checklist and scoring rubric available during the case to support consistent scoring and to ensure key teaching points weren’t missed. During the session, they were ready to gently redirect learners if they began role-playing dialogue or acting out the encounter, and instead, prompted them to list their intended actions, orders, and clinical decisions clearly and succinctly.

Of the residents, eight were PGY 1, and five were PGY 2. Overall, between both groups, the confidence level improved from 1.86 pre- to 2.92 post (*P*=0.005). PGY 1 residents had a lower confidence level starting out at an average of 1.5 with improvement to 2.63 (p=0.026) compared to PGY 2’s that started out at 2.4 and improved to 3.4 (p=0.089). The PGY 2 improvement was also not statistically significant.

Feedback from the session was overall positive. Most residents agreed the teaching was clear and concise and would like more on the topic (Figure 1). Some of the verbal feedback received included: “I thought this was great! I liked watching the video first so I was accustomed to the format, and I think it will be so helpful to do these going forward,” and “Great practice, I’m definitely early on so I need more exposure but very helpful.”

## Figures and Tables

**Table:** 

**HR:** 15	**BP:** 95/60	**RR:** 18	**Sat:** 87% on RA	**T:** 38.0°C	**Wt:** 50 kg

**Table:** 

**I. History**		**Yes**	**No**
**1a**	Asked about onset/duration of symptoms		
**1b**	Asked about past sickle cell crises or transfusion		
**2a**	Explained rationale for asking about prompt 2 #1		
**2b**	Explained rationale about asking about prompt 2 #2		
**II. Physical Examination**			
**3a**	Assessed for severe anemia or dehydration		
**3b**	Lung auscultation or oxygenation check for respiratory distress		
**4**	Explained rational for prompt 4		
**III. Differential Diagnosis**			
**5a**	Acute Chest Syndrome		
**5b**	Pneumonia		
**5c**	Acute Vaso-occlusive Crisis		
**IV. Diagnostic Studies**			
**6a**	Ordered labs including CBC, reticulocyte count and LDH		
**6b**	Ordered blood cultures and lactic acid		
**6c**	Ordered CXR		
**7a**	Provided rationale for answer to prompt 7 #1		
**7b**	Provided rationale for the answer to prompt 7 #2		
**V. Treatment and Other Actions**			
**8a**	Gave supplemental oxygen		
**8b**	Initiated empiric antibiotics		
**6c**	Displayed rationale for answer to prompt 9 #1		
**9a**	Displayed rationale for answer to prompt 9 #2		
**9b**	Gave supplemental oxygen		
**VI. Broad Principles**			
**10**	Discuss hypoxia worsens sickling causing overall worsening clinical status.		
**VII. Final Diagnosis**			
**11**	Acute chest syndrome		
**VIII. Disposition**			
**12**	Appropriate tele-bed admission		
**13**	Explained reasoning for hospital disposition		
**IX. Transitions of Care**			
**14a**	Acknowledged team sign-out		
**14b**	Explained diagnosis and plan to patient		

**Table:** 

Candidate Task Sheet	
**#1**	**Emergency Department Admitting Form**
**#2**	**Additional Historical Information**
**#3**	**Physical Exam Findings**
**#4**	**Diagnostic Studies: CXR**
**#5**	**Diagnostic Studies: Laboratory Results**
**#6**	**Diagnostic Studies: ECG**

**Table:** Clinical Decision-Making Candidate Task Sheet

CASE PARAMETERS
This is a 15-minute case You will interact with two examiners. This is an interview style without role playing; you should simply reply to the questions asked. You may be interrupted to move you through the case; this is not a reflection of your performance.
**PATIENT INFORMATION**
The patient will be a 32-year-old female with cough and fever with history of sickle cell.
**VITAL SIGNS**
BP: 95/60 mmHg, P: 116/min, R: 18/min, T: 38.0°C, 02 Sat: 87% on room air
**TASK STATEMENT**
Your tasks are as follows: List pertinent elements of a focused history and physical exam Develop an appropriate differential and/or provisional diagnosis Select and interpret appropriate studies Articulate appropriate patient management including discharge instructions

**STIMULUS 1 t6-jetem-11-4-ce1:** Emergency Department Admitting Form

Patient Information	
**Patient Name**	Brenda
**Age**	32-year-old
**Gender**	Female
**Method of Arrival**	Personal vehicle
**General Appearance/History of Present Illness**	Cough/fever with history of sickle cell
**Vital Signs on ED Arrival**	BP: 95/60 P: 115 beats per minute R: 18 respirations per minute T: 38° Celsius O2 sat: 87% on room air

**STIMULUS 2 t7-jetem-11-4-ce1:** Additional Historical Information

History of Present Illness/Description of Event	
Patient has had two days of cough and fever with a history of sickle cell. She has fever, nonproductive cough, right chest pain and shortness of breath associated. Currently she is complaining of worsened constant chest pain, shortness of breath and chills.	
**Past Medical History**	Sickle Cell and has been hospitalized for pain crisis.
**Medications**	Hydroxyurea, Oxycodone PRN, Folic Acid, Ibuprofen
**Allergies**	none
**Social History**	No tobacco, alcohol, or illicit drug use

**STIMULUS 3 t8-jetem-11-4-ce1:** Physical Exam Findings

Physical Examination	
**General**	uncomfortable
**HEENT**	conjunctival pallor, dry mucous membranes, otherwise normal
**Chest**	normal
**Respiratory**	patient moderately tachypneic, speaking in short sentences, moderate accessory muscle use, scattered rhonchi
**Cardiac**	tachycardic, systolic flow murmur, no rubs or gallops, S1, S2 heard, normal perfusion
**Abdominal**	normal
**Extremities**	normal
**Back**	paraspinal lumbar tenderness, no localizing pain, no step-off or deformity
**Neurologic**	normal
**Derm**	feels flushed and febrile
**Lymph**	normal

**STIMULUS 4:**
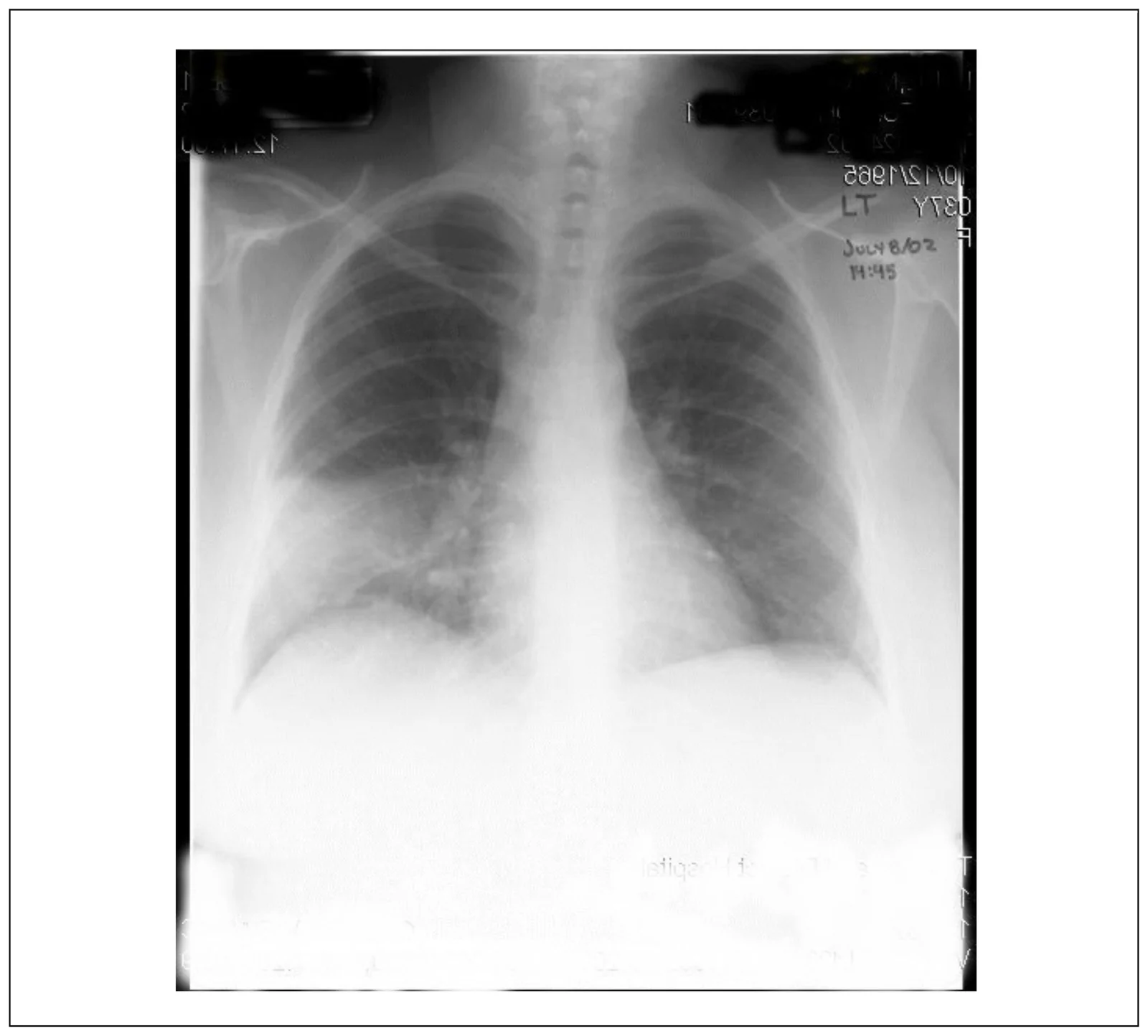
Diagnostic Studies: CXR^10^

**STIMULUS 5:** Laboratory Results

Reticulocytes	11.2%	

LDH	752	

CBC	WBC	15.6
	Hgb	8.55
	Hct	25.7
	Platelets	433
	Differential	
	Neutrophils	37%
	Lymphocytes	2.68
	Monocytes	0.89%
	Eosinophils	1.2
	HbF%	11.1

CMP	normal	

Pregnancy test	negative	

Troponin	12 ng/L	

D-Dimer	480 ng/L	

**STIMULUS 6:**
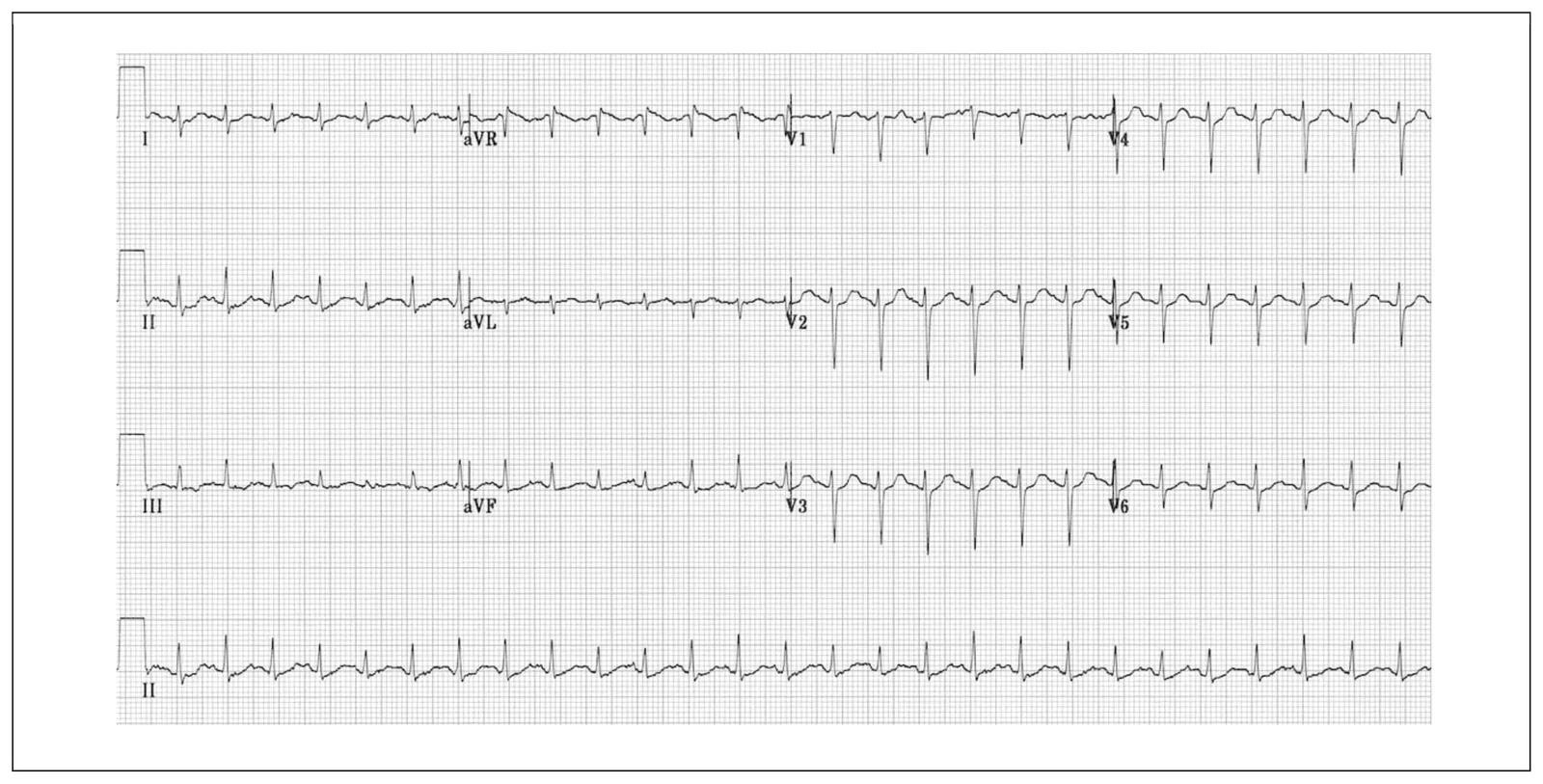
ECG^11^ Ventricular rate 120 BPM PR interval: 140 QRS duration: 80 MS QTc: 539 MS

**Figure 1:**
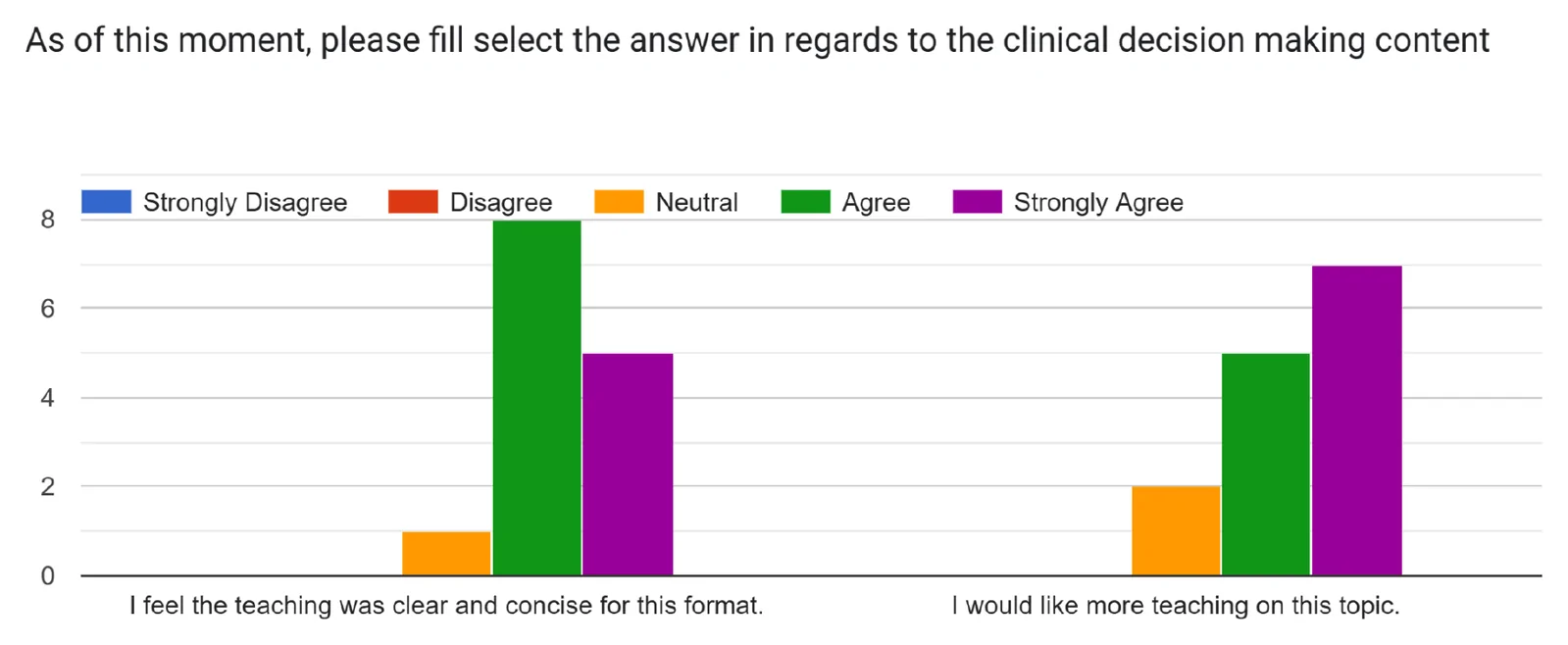


## FOR EXAMINER ONLY

### Clinical Decision-Making Case: Sickle Cell Crisis with Acute Chest Syndrome

**Diagnosis:** Acute Chest Syndrome

**Case Summary:** 32-year-old female with sickle cell anemia (HgbSS) presents with symptoms consistent with acute chest syndrome, supported by fever, cough, new pulmonary infiltrates on chest x-ray, and increased work of breathing with an oxygen requirement. Key management includes oxygen supplementation, careful (judicious) IV fluids, IV opioid analgesia, and antibiotics for community-acquired pneumonia coverage, with early hematology consultation given the risk of rapid decompensation. She should be admitted to a monitored setting with appropriate nursing support for close reassessment and escalation if needed. A patient who clinically deteriorates or requires exchange transfusion will require admission into a stepdown or ICU level bed.

**Synopsis of Physical Exam:**

- **General:** uncomfortable
- **HEENT**: conjunctival pallor, dry mucous membranes, otherwise normal
- Chest: normal
- **Lungs:** patient moderately tachypneic, speaking in short sentences, moderate accessory muscle use, scattered rhonchi
- **Heart:** tachycardic, systolic flow murmur, no rubs or gallops, S1, S2 heard, normal perfusion
- **Abdomen:** normal
- **Extremities:** normal
- **Back:** Paraspinal lumbar tenderness, no localizing pain, no step-off or deformity
- **Neuro:** normal
- **Skin:** feels flushed and febrile
- **Lymph:** normal

### Clinical Decision-Making Case: Sickle Cell Crisis with Acute Chest Syndrome Examiner Script

**Case Introduction:**

“Hello Doctor, this is a clinical decision-making case. Remember, there is no role playing. You should simply respond to the questions I ask. At times, you may be asked general questions about your clinical decision-making. Additionally, at times I may interrupt you to move you through the case; this is not a reflection of your performance. You will have 15 minutes to complete the case. Do you have any questions? All right, our time begins now.”

“The patient will be a 32-year-old female with cough and fever.”

Provide Learner Stimulus #1

#### HISTORY

Prompt 1:

“Here is the initial information regarding this patient. After you have read it, please give me a list of the additional pertinent historical information you would obtain.”

Prompt 2**:**

This response will depend on questions to the learner and will ultimately be chosen from examples below by the interviewer.

**ASK THIS PROMPT TWICE ABOUT TWO SEPARATE TOPICS**

Examples:

- “You indicated you would ask about the previous need for transfusion. Why is this important to you?”
- “You indicated you would ask about immunization status. Why is this important to you?”
- “You indicated that you would ask about her social history; is there anything specifically you would want to know, and why?”
- “You asked about her medication list; is there a specific medication that you are interested in?”
- “You asked about her past hospitalizations; was there a specific diagnosis or hospitalization you are interested in?”

Scoring Guidelines:

Rationale: Learners should ask when symptoms started and how long symptoms have been ongoing. Learners should ask about past sickle cell crises and if there has been a need for transfusions since this may change care. Learners should ask about past immunizations because sickle cell patients are at high risk for certain infections and should receive a special vaccination schedule for haemophilus influenzae type B, pneumococcal vaccine, and meningococcal vaccine. Learners should ask about medications that the patient is already on because narcotic medications and preventative medications are important treatments for a sickle cell patient.

General Guidelines:

- If candidate begins managing the case like a standard case, examiner states, “Remember Doctor, there is no role playing in this case. Please list the additional information you want to obtain.”
- If candidate does not offer a complete list of historical information, examiner should pause long enough to allow them to list additional items, before asking “why” questions.
- If candidate mentions “past medical history,” or “social history,” examiner clarifies by asking, “What specifically do you want to know about the past medical history/social history?”

Provide Learner Stimulus #2

“You are provided with the following additional historical information.” Show the historical information provided on Learner Stimulus #2.

#### PHYSICAL EXAMINATION

“You are provided with the following additional historical information:

A 32-year-old female with a history of sickle cell anemia (HgbSS) presents with two days of cough and fever. Her cough is described as non-productive and associated with shortness of breath. She has had limited PO intake x2 days. She has pain on the right side of her chest described as achy and worsened when she takes a deep breath or cough but has now become constant over the past couple hours. She was last hospitalized five months prior for a pain crisis. Review of systems (ROS) is otherwise negative. She moved states about 2 months ago and has been out of her medications since due to lack of hematology follow-up and medical insurance.”

Prompt 3:

“Based on what you now know, please give me a list of specific physical examination findings you would be looking for.”

Prompt 4:

This response will depend on learner’s questions and will ultimately be chosen from examples below by the interviewer.

Examples:

- “You examined the lungs. How does that help you in this case?”
- “You evaluated her heart; what would you be concerned for in this patient?
- “You looked at her mucous membranes; what would you want to be looking for on this exam?”
- “You looked at her extremities; what would you want to be looking for on this exam?”

Scoring Guidelines:

Rationale: Learners should be looking at the vital signs of fever, tachycardia, hypoperfusion and increased respiratory rate as a concern for septic shock. They should be evaluating for signs of severe anemia or dehydration by looking at mucous membranes for pallor and dryness. They should be listening to the lungs for signs of respiratory distress, or crackles/ronchi or wheezes that would indicate an infection or edema. They should be evaluating heart sounds and circulation for signs of significant heart murmur or shock.

“You are provided with the following physical exam findings:”

Provide Learner Stimulus #3

#### DIFFERENTIAL DIAGNOSIS

Prompt 5:

“Based on what you now know, what are the top three items on your differential diagnosis based on the most likely conditions?” (If more than three conditions are mentioned, say, “OK, thank you. Please give me your three, and only three, most likely diagnoses.”)

Prompt 5:

“Based on what you now know, what are the top three items on your differential diagnosis based on the most likely conditions?” (If more than three conditions are mentioned, say, “OK, thank you. Please give me your three, and only three, most likely diagnoses.”)

Appropriate differential diagnoses include:

- Acute chest syndrome
- Pneumonia
- Vaso-occlusive crisis
- Pulmonary embolism
- Asthma Attack
- Aplastic Anemia

Scoring Guidelines:

Rationale: With regards to pathologies related to sickle cell, learners should recognize that acute chest syndrome is the leading life-threatening concern for this sickle cell patient. An acute Vaso-occlusive crisis in general is also a leading concern. Given the vital signs and history, they should also be concerned for pneumonia and /or sepsis from pneumonia in a patient with respiratory distress, fever, and hypoxia. Other differentials unrelated to sickle cell could include pulmonary embolus or asthma attack but are far less likely given the history and physical exam.

#### DIAGNOSTIC STUDIES

Prompt 6:

“Based on what you know and your working differential diagnosis, what, if any, diagnostic studies would you order?”

Prompt 7:

This response will depend on learner’s questions and will ultimately be chosen by the interviewer from the list below.

**ASK THIS PROMPT TWICE ABOUT TWO SEPARATE TOPICS**

Examples:

- “Doctor, you ordered Reticulocyte count. Why did you order the study?”
- “You ordered blood cultures; what are you looking to rule out?”
- “You asked for a CXR; what would you be looking for specifically?”
- “You asked for CT imaging; would you want it with contrast or without, and why?”

Scoring Guidelines:

Rationale: Diagnostic studies evaluating Sickle Cell. To evaluate for acute vaso-occlusive crisis, learners should order a CBC, reticulocyte count and lactate dehydrogenase (LDH) test. Since the patient also has vital signs concerning for sepsis, learners should order blood cultures and lactic acid. Given the hypoxia and sickle cell history, learners should order a CXR and consider CT imaging to diagnose acute chest syndrome. Learners should be able to explain their rationale for ordering specific laboratory tests and imaging.

After the candidate responds,

Provide Learner Stimulus 4, 5 and 6 depending on what the learner ordered.

#### TREATMENT AND OTHER ACTIONS

Prompt 8:

“Based on what you now know, what treatments, if any, would you order and/or what actions, if any, would you perform?”

Prompt 9:

“Doctor, you ordered Rocephin and Azithromycin. Why did you administer antibiotics?”

“You ordered fluids; why did you order fluids?

“You ordered pain medication; why did you order pain medication?

**ASK THIS PROMPT TWICE ABOUT TWO SEPARATE TOPICS**

Scoring Guidelines:

Rationale: Treatment for Sickle Cell Anemia includes broad spectrum antibiotic coverage for pneumonia, a fluid bolus for dehydration/sepsis, pain medication including but not limited to Toradol, Tylenol, and narcotics, and supplemental oxygen.

#### BROAD PRINCIPLES

Prompt 10:

“Not specific to this case, doctor, but what are the key concepts that guide early management of acute chest syndrome in sickle cell disease—especially oxygenation, antibiotics, fluids, pain control/incentive spirometry, and when transfusion is indicated—and what is the reasoning behind them?

Scoring Guidelines:

Rationale: Scoring is based on whether the learner identifies that acute chest syndrome can deteriorate rapidly and therefore requires early intervention and close monitoring. The learner should explain that hypoxia worsens sickling and should be treated promptly with oxygen (with escalation of respiratory support as needed). Empiric antibiotics with community-acquired pneumonia coverage including atypicals should be ordered because infection is a common trigger and difficult to exclude initially. Learners should emphasize incentive spirometry and adequate pain control to prevent splinting and atelectasis and to improve ventilation and note that IV fluids should be given judiciously to avoid both dehydration and pulmonary overload. Exchange transfusion is a key escalation strategy to improve oxygen delivery and reduce HbS burden (with exchange transfusion reserved for severe or worsening cases) and should be ordered after a hematology consult if the patient is deteriorating, specifically with rapidly progressive respiratory failure, worsening neurologic status, or multi-organ involvement.

#### FINAL DIAGNOSIS

Prompt 11:

“Based on everything you know about this case, what is your final diagnosis?”

Acute chest syndrome

Scoring Guidelines:

Rationale: Verbalizing acute chest syndrome and/or vaso-occlusive crisis meets the critical action. If the candidate mentions something vague such as pneumonia with hypoxia or sepsis (usually a physical exam finding or imaging finding instead of diagnosis), the examiner asks, “Can you be more specific about the diagnosis and how this relates to sickle cell disease?”

#### DISPOSITION

Prompt 12:

“Based on what you know, what should be the disposition of this patient?”

Prompt 13:

“Why would you [admit/discharge] this patient?”

Scoring Guidelines:

Rationale: Verbalizing a disposition and level of care and explaining it is necessary for acute chest syndrome. The patient would require admission for continuation of supplemental oxygen, and IV analgesia. The patient would require tele-bed level monitoring given the concern for decompensation and potential need for transfusion. The patient would require stepdown or ICU level care if they fail initial therapy or require exchange transfusion as evidenced by persistent uncontrolled chest pain, hypoxia, or continued respiratory distress.

#### TRANSITION OF CARE

Prompt 14:

“What specific actions would you take at the time of [admission/discharge]?

Scoring Guidelines:

Rationale: Verbalizing final actions such as explaining the diagnosis to the patient and family, dispositioning the patient to a monitored bed, and re-evaluation of pain levels, hydration status and oxygenation.

*Thank you, Doctor. That concludes this case. Please tear up your notes* [Table t6-jetem-11-4-ce1] [Table t7-jetem-11-4-ce1] [Table t8-jetem-11-4-ce1]

## CERTIFYING EXAM ASSESSMENT

*Sickle Cell Crisis with Acute Chest Syndrome*

Learner: _________________________________________

**Summative and formative comments:**

## DEBRIEFING AND EVALUATION PEARLS

### Clinical Decision-Making Case: Sickle Cell Crisis with Acute Chest Syndrome

#### Diagnosis of ACS

- Defined by a new pulmonary infiltrate on chest imaging plus one or more of the following: fever, chest pain, cough, dyspnea, or hypoxia.^1^
- Common triggers include infection, fat embolism, infarction, or hypoventilation.^1^

#### Initial Evaluation

- Key history: frequency of sickle cell crises, prior ACS episodes, home oxygen or transfusions, immunization status, recent infections, baseline oxygen saturation, outpatient hematologist. 4
- The physical exam should focus on first addressing ABC’s to ensure patient stability, with particular focus on respiratory status, including signs of distress, breath sounds, and oxygen saturation. In this case, this will require use of supplemental oxygen. Once this is addressed, physicians should perform a focused physical examination, with particular attention to pulmonary exam. 7

#### Key Diagnostics

- Laboratory tests: CBC with differential, reticulocyte count, haptoglobin, consider arterial blood gas (ABG), beta-HCG, blood cultures.^9^
- Imaging: Chest X-ray (often shows lobar or multilobar infiltrates); consider D-Dimer or CTA chest if pulmonary embolism is suspected.^9^

#### Management Priorities

- Administer supplemental oxygen to maintain saturation ≥95%.^8^
- Start empiric antibiotics such as intravenous ceftriaxone and oral macrolide.^8^,^9^
- Provide IV fluids but re-evaluate for over-hydration which can cause pulmonary edema.^9^
- Treat pain appropriately with opioids.
- Once pain is controlled, encourage use of incentive spirometry to reduce hypoventilation.^8^.^9^
- Consult hematology early for potential transfusion (simple or exchange).^9^,^10^

#### Disposition

- Admit to a monitored bed at minimum because clinical deterioration can occur rapidly and unexpectedly.^1^ However, ICU admission should be considered in sickle cell patients with acute chest syndrome who require exchange transfusion, have hypoxemic respiratory distress, tachypnea, hypotension, acute kidney injury, pulmonary hypertension, or need respiratory support. Stable patients without organ failure may be managed on monitored inpatient units/telemetry depending on institutional capability.^10^
- Ensure clear documentation, handoff, and education on expected course and follow-up needs.

#### Feedback Tips for Examiner

- Did the learner identify ACS early based on clinical features and imaging?
- Did they ask for a focused and complete history and perform a targeted physical exam?
- Were the diagnostic tests appropriate and prioritized logically?
- Did the management plan reflect current evidence and urgency?
- Was the disposition appropriate, with clear rationale and transition of care?
